# Comparative genomic and evolutionary analysis of stress-associated proteins in *Prunus persica* and distinct plant species under abiotic stress

**DOI:** 10.3389/fgene.2026.1821359

**Published:** 2026-05-12

**Authors:** Wendou Zhang, Zamri Zainal, Nurulhikma Md Isa

**Affiliations:** 1 Department of Biological Sciences and Biotechnology, Faculty of Science and Technology, Universiti Kebangsaan Malaysia, Bangi, Selangor, Malaysia; 2 O. Peach Engineering Tech. Research Center, Changde Vocational and Technical College, Changde, China

**Keywords:** A20/AN1 zinc finger domains, abiotic stress response, cis-regulatory elements, gene family, *Prunus persica*, RNA-seq profiling, stress-associated proteins (SAPs), synteny and duplication

## Abstract

Stress-Associated Proteins (SAPs) are important zinc-finger proteins containing A20 and/or AN1 domains and are widely involved in plant stress responses and hormone signaling. However, the SAP gene family in peach (*Prunus persica*) has not yet been systematically characterized. In this study, we performed a genome-wide identification and comparative analysis of the *PpSAP* family to investigate its evolutionary relationships, structural features, promoter characteristics, and stress-responsive expression patterns. A total of nine *PpSAPs* (*PpSAP1-PpSAP9*) were identified and classified into four phylogenetic clades, providing a framework for understanding SAP family diversification in peach and other representative plant species. Comparative analysis showed that the SAP gene family is relatively conserved within *Rosaceae* species but exhibits greater divergence in more distantly related species, such as *Arabidopsis thaliana* and *Oryza sativa*, suggesting lineage-specific evolutionary differentiation. Promoter analysis revealed that *PpSAPs* contain abundant cis-regulatory elements associated with stress responses and hormone signaling, including ABA, GA, MYB, and anaerobic-responsive elements. RNA-seq expression profiling further demonstrated that *PpSAPs* exhibit diverse transcriptional responses under drought, salinity, ethylene, and cold stress conditions. In particular, different *PpSAP* members displayed distinct temporal expression patterns during cold treatment, indicating potential functional divergence within the family. Overall, this study provides the first comprehensive genomic and evolutionary characterization of the *PpSAP* gene family in peach and identifies several stress-responsive candidate genes for future functional validation and peach stress-resilience improvement.

## 1 Introduction

Peach (*Prunus persica*) is a major temperate fruit crop whose growth, yield stability, and fruit quality are frequently constrained by abiotic stresses such as drought, salinity, and low temperature. Improving stress resilience in perennial fruit trees therefore requires the identification of regulatory genes that integrate multiple environmental and hormonal signals while minimizing growth penalties.

SAPs are zinc-finger proteins characterized by A20 and/or AN1 domains and play crucial roles in plant stress responses. SAPs have been experimentally validated as functional regulators in various species. For instance, the *LcSAP* in grass species (A20-AN1 SAP) enhances salt tolerance when expressed in yeast, providing early functional evidence for SAP involvement in cellular salt tolerance (Liu et al., 2017). In halophytes, *SAPs* confer tolerance to extreme environments: *LmSAP* improves salt and ionic tolerance in transgenic tobacco and yeast (Ben Saad et al., 2018), while *ThSAP6* enhances salt tolerance by boosting antioxidant capacity and reducing membrane damage (Zhao et al., 2021). In woody species, *PtSAP13* is induced by salt stress, and its overexpression enhances salt tolerance, with increased antioxidant enzyme activities and activation of stress-related pathways (Li et al., 2019). These studies collectively demonstrate that *SAPs* are widely conserved stress regulators, spanning herbaceous plants to woody perennials.

SAPs function as key regulators in abiotic stress responses, with mechanisms involving the ubiquitin-proteasome system and hormonal signaling pathways. For example, TaSAP5 from wheat functions as an E3 ubiquitin ligase, promoting DRIP protein degradation and enhancing DREB2A abundance, thereby improving drought tolerance and yield under severe drought conditions (Zhang et al., 2017). In orchids, Pha13 (and its *Arabidopsis* homolog AtSAP5) is a key component of salicylic acid (SA)-mediated antiviral immunity, displaying E3 ligase activity and ubiquitin-chain binding, with the A20 domain being crucial for immune regulation (Chang et al., 2018). Analysis of LmSAP’s A20 domain revealed its essential role in tolerance to salt and osmotic stress, highlighting A20’s significance in SAP regulation (Ben Saad et al., 2019). SAPs are also involved in ABA-related responses: OsSAP8 modulates salt and osmotic stress tolerance, displaying altered ABA-related phenotypes in *Arabidopsis*, while OsSAP10 enhances water-deficit tolerance through proteasome-associated interactions and positive regulation of ABA signaling (Vashisth et al., 2024). Importantly, SAP functions can be context-dependent, with some SAPs involved in stress-growth trade-offs: PagSAP1 in poplar increases salt tolerance when downregulated (Yoon et al., 2018), whereas AdhSAP4 in kiwi negatively regulates salinity tolerance (Parra et al., 2025).

In *Prunus* species, functional evidence suggests a dual role for SAPs in both stress response and development. For example, PpSAP1 in peach interacts with polyubiquitin proteins, and its overexpression in plums enhances water retention under drought stress, while altering leaf morphology and transcriptionally repressing cell growth-related pathways. These findings suggest that SAPs coordinate water retention and growth through TOR-associated regulation (Lloret et al., 2017). Together, these observations identify SAPs as promising regulators of stress resilience in fruit trees and highlight the need for a systematic, peach-specific characterization of this family. Nevertheless, although SAP genes have been studied in several annual crops and some woody plants, an integrated analysis of peach SAP genes encompassing evolutionary relationships, promoter architecture, and stress-responsive transcription remains unavailable. This gap hinders the prioritization of candidate genes for functional validation and breeding-oriented improvement of abiotic stress resilience in perennial fruit crops.

Accordingly, we performed a genome-wide identification and comparative evolutionary analysis of the SAP gene family in peach, integrating phylogenetic relationships across representative plant species, domain organization, gene structure, chromosomal distribution, duplication history, promoter cis-element profiling, and stress-responsive expression patterns.

## 2 Materials and methods

### 2.1 Genome resources and SAP reference sequences

The peach (*Prunus persica*) reference genome assembly and annotation files (FASTA and GFF3) were downloaded from the Genome Database for Rosaceae (GDR) and corresponded to the Prunus persica ‘Zhongyoutao 14’ Genome v1.0 Assembly and Annotation (Lian et al., 2022). This assembly was generated using PacBio long-read sequencing data and constructed with Canu v1.9. The retrieved dataset included the genome sequence, CDS sequences, predicted protein sequences, and the corresponding GFF3 annotation files. To minimize coordinate and feature inconsistencies, the same genome version (v1.0) was used throughout all downstream analyses. Arabidopsis SAP proteins (AtSAP1-AtSAP14) were compiled according to (Vij and Tyagi, 2006). For alignment visualization, representative AtSAP sequences (AtSAP1 and AtSAP2) were obtained from TAIR (Berardini et al., 2015).

### 2.2 Identification of SAP family members in peach and basic characterization

SAP candidates were identified using a combined homology-based and HMM-based pipeline implemented in TBtools v2.376 (Chen et al., 2023). For the homology search, Arabidopsis SAP proteins were used as queries to perform BLASTP against the peach proteome using the “Blast Compare Two Seqs [Sets]” module in TBtools (NumofThreads = 2; E-value = 1e^−5^; NumofHits = 500; NumofAligns = 250). Hits were filtered with an E-value cutoff of 1e^−5^, and the top-scoring matches were retained and merged with the HMM-derived candidates (Camacho et al., 2009). In parallel, HMMER searches were conducted using the TBtools “Advanced HMMER Search” module with Pfam profiles corresponding to the A20-type zinc finger (PF01754; zf-A20) and the AN1-type zinc finger (PF01428; zf-AN1) from Pfam v32.0 (Mistry et al., 2021). The Pfam accessions were entered directly into TBtools to call the corresponding domain models.

Additionally, InterProScan (version 5.47–82.0) was used for further domain validation. InterProScan integrates multiple domain databases, including Pfam, SMART, ProDom, and TIGRFAMs, to provide a more comprehensive assessment of domain presence across protein sequences. For the identification of SAP genes, proteins were considered as canonical SAPs if they contained at least one of the diagnostic A20 or AN1 domains. To ensure high-confidence domain assignments, E-value cutoffs were set to 1e^−5^ for all domain matches. Only genes that exhibited significant domain matches to A20 (PF01754) and/or AN1 (PF01428) were retained in the final SAP gene set. Proteins with partial or weak domain evidence, such as those lacking the full A20 or AN1 zinc-finger motif or showing low alignment confidence, were excluded from the final selection.

InterProScan v5.47-82.0 was subsequently used for domain validation. Proteins containing at least one confidently supported A20 and/or AN1 domain were retained as SAP candidates. Sequences lacking complete or well-supported A20/AN1 domains were excluded from the final SAP gene set. Protein physicochemical properties, including sequence length, molecular weight, theoretical isoelectric point, instability index, aliphatic index, and GRAVY, were calculated using the protein analysis utilities in TBtools v2.376. Subcellular localization was predicted with DeepLoc 2.1 under default settings. DeepLoc 2.1 reports one or more likely subcellular compartments and membrane association types; for each protein, the most probable compartment(s) and membrane type, when applicable, were recorded (Ødum et al., 2024; Thumuluri et al., 2022).

### 2.3 Multiple sequence alignment, phylogenetic inference, and conserved domain annotation

Protein sequences of the nine PpSAPs and fourteen *Arabidopsis* SAPs (AtSAPs) were aligned using MAFFT as implemented in TBtools v2.376 with default parameters. Based on the resulting alignment, a maximum-likelihood (ML) phylogeny was inferred with IQ-TREE using the JTT + G model selected by ModelFinder. Branch support was evaluated using the ultrafast bootstrap method with 5,000 replicates (Minh et al., 2013). The inferred tree was visualized and annotated in iTOL (Letunic and Bork, 2021).

Conserved domains in PpSAPs were annotated using the NCBI Conserved Domain Database (CDD) Batch CD-Search under default settings, and only domains meeting the default reporting thresholds were retained (Lu et al., 2020). Domain architecture was used for final classification of PpSAPs into A20-AN1 type or AN1-only type, with proteins carrying two AN1 domains designated as the AN1 × 2 subtype. Multiple sequence alignment figures for AtSAP1, AtSAP2, and PpSAPs were generated with ESPript (Robert and Gouet, 2014).

### 2.4 Gene structure visualization, motif discovery, and integrated plot construction

Gene structures were parsed from the peach GFF3 annotation file and visualized using the Gene Structure View (Advanced) module in TBtools v2.376. Conserved motifs were identified with MEME Suite v5.3.0 using the following parameters: maximum number of motifs = 10, minimum motif width = 6, and maximum motif width = 50 (Bailey et al., 2015). Motifs were ranked by MEME, and the top-scoring motifs within the specified width range were retained for cross-gene comparisons.

To facilitate side-by-side comparisons among clades, phylogenetic relationships, MEME-derived motif distributions, CDD-based domain annotations, and gene structures were integrated into a composite visualization in TBtools v2.376. Panels were arranged and aligned in the order of phylogeny, motifs, domains, and gene structure to enable direct structural and evolutionary comparisons across PpSAP subgroups.

### 2.5 Chromosomal distribution, duplication/collinearity, interspecies synteny, and Ka/Ks estimation

Chromosomal coordinates of PpSAP1-PpSAP9 were obtained by parsing the “gene” features in the peach GFF3 annotation, including chromosome or scaffold identifiers and gene start/end positions, and were visualized in TBtools v2.376. To detect intragenomic collinearity and assign duplication types, an all-versus-all protein similarity search was performed, and the resulting homologous matches were provided to MCScanX v1.0 for collinearity scanning using an E-value cutoff of 1e^−10^. Duplication categories, including WGD/segmental, tandem, proximal, and dispersed duplication, were assigned following standard MCScanX conventions (Wang et al., 2012).

For interspecies synteny analyses, genome assemblies and corresponding gene annotations for six representative species (*Prunus dulcis, Prunus mume, Malus domestica, Fragaria × ananassa, Arabidopsis thaliana, and Oryza sativa*) were collected from public repositories; data sources and versions are provided in Supplementary Table S5. Pairwise synteny between peach and each comparator genome was inferred using the MCScanX-based pipeline implemented in TBtools v2.376 with an E-value cutoff of 1e−10. Collinear blocks and syntenic gene pairs were identified genome-wide, and SAP-related syntenic relationships were extracted by anchoring on *PpSAP1-PpSAP9*. Dual synteny plots were generated using the Dual Synteny Plot module in TBtools v2.376. To improve readability and maintain consistency with the Results, displayed links were restricted to syntenic orthologous pairs anchored by *PpSAP1-PpSAP9*, whereas chromosome (or pseudochromosome) backbones were shown at the whole-genome scale.

For Ka/Ks estimation of duplicated *PpSAPs* gene pairs, coding sequences (CDSs) were retrieved from the reference annotation. Codon-preserving alignments were generated by mapping protein alignments back to the corresponding CDS sequences to obtain codon-aware CDS alignments. Ka, Ks, and Ka/Ks values were then calculated using the codon-based Ka/Ks workflow implemented in TBtools. Comparisons with saturated or undefined synonymous substitution estimates, such as extremely high Ks values or missing Ks values, were retained for describing divergence but were excluded from selective-pressure inference.

### 2.6 Promoter cis-element analysis and RNA-seq expression profiling

Promoter regions were defined as the 2-kb upstream sequences relative to the annotated translation start site (ATG). Promoters were extracted using the GXF Sequences Extract function in TBtools v2.376 with an initialized annotation, selecting “Parent” and “CDS,” setting the upstream length to 2,000 bp, and enabling “Retain Only UpStream or DownStream Bases.” Promoter extraction was performed in a strand-specific manner. Loci located within 2 kb of chromosome or scaffold ends were automatically truncated according to sequence availability. FASTA headers were simplified using ID Simplify, and promoter sequences were retrieved using the recommended Fasta Extract function.

Cis-acting regulatory elements were predicted using PlantCARE with default setting (Lescot et al., 2002). PlantCARE tabular outputs were curated by retaining the gene ID, element start position, element length, and annotation. Non-informative entries were removed, and element names were grouped into concise categories for plotting. Promoter lengths were obtained using Fasta Stats with “Keep Only Sequence Length” enabled. Cis element count heatmaps and positional distribution plots were generated using Basic Biosequence View in TBtools. To improve readability in positional plots, element spans were uniformly expanded before visualization by adjusting coordinates (start-10 bp; length +10 bp).

For expression profiling, public RNA-seq datasets for peach abiotic and hormone treatments were retrieved from NCBI SRA; run accessions are listed in Supplementary Table S4. SRA metadata were downloaded into Full XML format and converted to tabular files using the SRA XML to Table function in TBtools to curate sample groups and biological replicates. SRA runs were downloaded and converted to FASTQ files using the TBtools Pro plugin (SRA to Fastq). Expression was quantified using kallisto v0.46.1 in pseudo-alignment mode with default settings. The kallisto reference index was built by extracting exon-based sequences from the peach annotation using GXF Sequences Extract (Parent + exon). Non-chromosomal or unanchored sequences were removed when necessary.

Transcript abundance was represented as TPM (Transcripts Per Million). For NaCl, drought, and ethylene treatments, expression levels of *PpSAPs* were visualized as bar charts using mean TPM values of three biological replicates, and the data are presented as mean ± SD of three biological replicates. Statistical differences between each treatment and its corresponding control were evaluated using a two-tailed Student’s t-test, and differences were considered significant at p < 0.05. For cold stress, time-course expression profiles were visualized as line plots using log2 (TPM +1) values from three biological replicates, and the data are presented as mean ± SD of three biological replicates. Treatment responsiveness was additionally summarized for descriptive comparison using an empirical cutoff of |log2FC| > 1, where log2FC was calculated from mean TPM values of treatment and matched control groups. Standardized TPM heatmaps with log scaling and row-wise scaling were retained in Supplementary Figure S1 to provide an overview of global expression patterns.

## 3 Results

### 3.1 Identification, phylogenetic classification and nomenclature of PpSAPs

**FIGURE 1:**
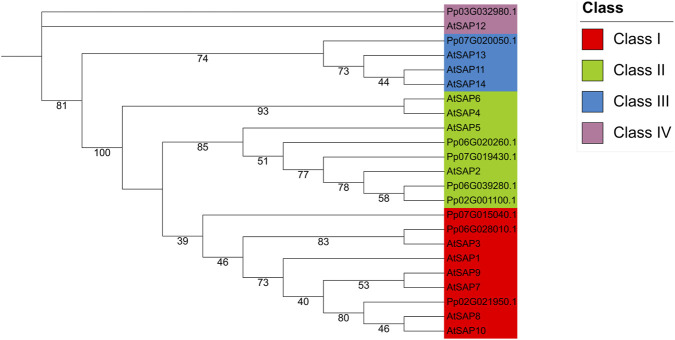
Phylogenetic classification of SAPs in peach and *Arabidopsis*. Maximum-likelihood phylogenetic tree constructed using protein sequences of 9 PpSAPs and 14 AtSAPs. Branch colors indicate the four clades (Class 1-Class 4). Peach members are labeled with PpSAP1-PpSAP9, and *Arabidopsis* members with AtSAP. Bootstrap support values are shown on major nodes.

SAP-like candidates were retrieved from the *Prunus persica* proteome using a combined homology-based search and Pfam HMM profiling, and the resulting set was further curated by domain validation based on InterPro annotations. After this two-step filtering and removal of redundancy, nine genes were retained as the curated PpSAP family (PpSAP1-PpSAP9). The retained proteins were supported by the presence of the diagnostic A20 (PF01754) and/or AN1 (PF01428) domains (Figure 1; Table 1).

**TABLE 1:** PpSAP family members and their phylogenetic classification predicted subcellular localization, and physicochemical properties.

Gene	Gene ID	Class	Type	Length (aa)	MW (kDa)	pI	Instability	Aliphatic	GRAVY	Localization
*PpSAP1*	*Pp02G001100.1*	2	A20-AN1	172	18.48	7.95	24.78	67.62	−0.458	Cytoplasm and nucleus
*PpSAP2*	*Pp06G028010.1*	1	A20-AN1	162	18.06	8.81	57.49	57.28	−0.609	Cytoplasm and nucleus
*PpSAP3*	*Pp07G015040.1*	1	A20-AN1	183	19.56	7.95	58.08	67.16	−0.2	Cytoplasm and nucleus
*PpSAP4*	*Pp02G021950.1*	1	AN1	202	21.69	6.32	52.57	59.9	−0.824	Cytoplasm and nucleus
*PpSAP5*	*Pp06G039280.1*	2	A20-AN1	173	18.48	7.55	29.78	59.19	−0.431	Cytoplasm and nucleus
*PpSAP6*	*Pp07G019430.1*	2	AN1	131	14.13	8.42	31.16	66.87	−0.34	Cytoplasm and nucleus
*PpSAP7*	*Pp06G020260.1*	2	A20-AN1	168	18.82	8.98	38.29	55.12	−0.87	Cytoplasm and nucleus
*PpSAP8*	Pp07G020050.1	2	AN1-AN1	292	31.98	8.72	44.31	59.45	−0.575	Cytoplasm
*PpSAP9*	Pp03G032980.1	4	AN1-AN1	192	21.62	9.01	45.95	48.18	−0.751	Cytoplasm

Protein length, molecular weight (MW), theoretical isoelectric point (pI), instability index, aliphatic index, and GRAVY were calculated using the protein analysis module in TBtools v2.376. Subcellular localization was predicted using DeepLoc 2.1 (DTU Health Tech) under default settings.

Three loci initially recovered by BLAST were excluded during domain validation for distinct reasons. Pp01G048020.1 showed only weak AN1-like similarity and did not receive consistent support as a canonical AN1-type zinc-finger across domain evidence; therefore, it was removed from the final set. In addition, Pp02G001090.1 and Pp07G020030.1 encoded unusually short open reading frames and lacked convincing A20/AN1 domain signatures, suggesting that they do not represent complete, canonical SAP proteins.

A maximum-likelihood phylogeny constructed from the protein sequences of the nine PpSAPs and fourteen *Arabidopsis* SAPs (AtSAPs) resolved four major clades (Class 1-Class 4) (Figure 1). Peach members were named PpSAP1-PpSAP9 based on their phylogenetic positions and the closest Arabidopsis homologs (Table 1). PpSAP1, identified by (Lloret et al., 2017), corresponds to the gene Pp02G001100.1 in this study. Others were named according to their phylogenetic relationships with Arabidopsis SAPs, and gene IDs are mapped in Table 1.

Physicochemical profiling indicated that PpSAP proteins range from 131 to 292 amino acids, with predicted molecular weights of 14.13–31.98 kDa (Table 1). Theoretical isoelectric points (pI) varied from 6.32 to 9.01, suggesting that both acidic and basic proteins are represented within the family. All members exhibited negative GRAVY values (−0.87 to −0.20), consistent with an overall hydrophilic character. Subcellular localization predictions further suggested that most PpSAPs (7/9) are distributed across both the cytoplasm and nucleus, whereas PpSAP8 and PpSAP9 were predicted to localize predominantly to the cytoplasm (Table 1). This distribution pattern is compatible with a role for most PpSAPs in linking cytoplasmic signaling processes to nuclear regulatory functions.

### 3.2 Multiple sequence alignment and conserved domain features of PpSAPs

**FIGURE 2:**
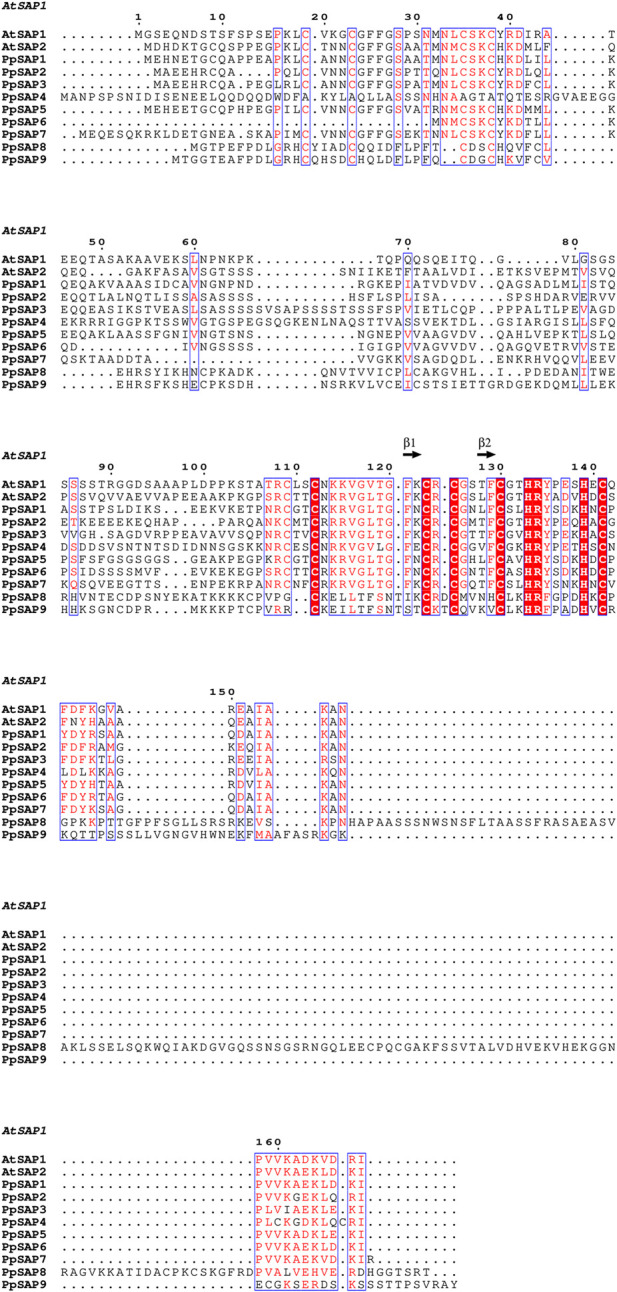
Multiple sequence alignment of peach PpSAPs with representative AtSAPs. Three conserved regions are indicated: CR1, the A20-associated cysteine framework; CR2, the AN1 core region; and CR3, the C-terminal signature segment. Conserved residues and lineage-associated divergence patterns among PpSAP members are highlighted.

To assess sequence conservation and domain organization within the peach SAP family, multiple sequence alignments were generated for PpSAP1-PpSAP9 together with two representative Arabidopsis proteins, AtSAP1 and AtSAP2 (Figure 2). Overall conservation was concentrated in the zinc-finger core regions corresponding to the A20 and/or AN1 domains, whereas flanking regions showed greater variability. In particular, higher divergence was observed toward the protein termini—most prominently at the C-terminus—suggesting lineage-specific remodeling superimposed on a conserved structural scaffold.

Within A20-containing PpSAPs, a canonical A20-associated cysteine framework (CR1) was apparent near the N-terminus and was characterized by conserved Cys residues (with occasional His substitutions) consistent with an A20-type zinc-finger architecture (Figure 2). A conserved AN1 core region (CR2) was also detected in the central portion of the alignment, with zinc-finger-associated residues being retained across members. By contrast, the C-terminal signature segment (CR3) exhibited comparatively higher sequence heterogeneity; however, short conserved patches were shared among subsets of PpSAPs, implying potential subgroup-specific regulatory features or functional specialization. Collectively, the conserved A20/AN1-related zinc-finger framework observed in peach closely mirrored that of AtSAP proteins, supporting evolutionary conservation of SAP-associated regulatory functions (Figure 2).

### 3.3 Gene structure and conserved motif analysis of *PpSAPs*

**FIGURE 3:**
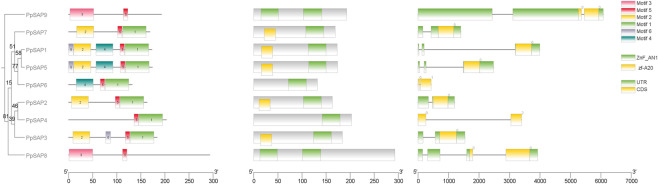
Integrated phylogeny, motif composition, conserved domains, and gene structures of PpSAPs. From left to right, the panel shows the phylogenetic relationships of PpSAPs, MEME-identified motifs (Motifs 1–7), CDD-predicted conserved domains (zf-A20 and ZnF-AN1), and exon-intron structures, enabling direct comparison of structural features across clades.

To investigate the evolutionary relationships and structural features of peach SAP genes, we integrated phylogenetic relationships, motif organization, conserved domains, and exon-intron structures of the nine *PpSAPs* (Figure 3). In the peach-only phylogeny, *PpSAPs* were resolved into two higher-order lineages, within which two robust sister relationships were consistently recovered: *PpSAP5* clustered with *PpSAP6*, and *PpSAP3* clustered with *PpSAP4* (Figure 3). Notably, *PpSAP8* and *PpSAP9* displayed more divergent structural signatures in the integrated view, suggesting greater subgroup-specific structural specialization than other *PpSAP* members.

Motif composition was broadly consistent with phylogenetic grouping. Except for *PpSAP8* and *PpSAP9*, most *PpSAPs* retained a conserved C-terminal motif pattern represented by the Motif 5-Motif 1 combination, supporting the presence of a shared motif backbone across the family (Figure 3). In contrast, *PpSAP8* and *PpSAP9* were dominated by a Motif 3 + Motif 5 configuration and lacked Motif 1, indicating a distinct motif architecture. Divergence was also evident within lineages: *PpSAP1* and *PpSAP5* exhibited identical motif architectures, whereas *PpSAP6* showed a simplified arrangement (Motif 4-Motif 5-Motif 1). In addition, *PpSAP7* and *PpSAP2* shared a stable Motif 2-Motif 5-Motif 1 pattern, consistent with stronger conservation of their core motif framework.

Gene structure analysis revealed substantial variation in exon number and gene span, ranging from <0.5 kb to ∼6.6 kb, with 1–4 exons across the family (Figure 3). Most *PpSAPs* displayed relatively compact structures: *PpSAP7*, *PpSAP2*, and *PpSAP3* each contained two exons, whereas *PpSAP5* contained three exons. *PpSAP1* showed a larger genomic span (∼4.0 kb), which was mainly attributable to intron expansion. By contrast, *PpSAP6* represented the shortest gene (<0.5 kb), whereas *PpSAP9* exhibited the largest span (∼6.6 kb), likely reflecting expansion of non-coding regions, including annotated UTRs.

### 3.4 Chromosomal distribution, duplication events and synteny of *PpSAPs*

**FIGURE 4:**
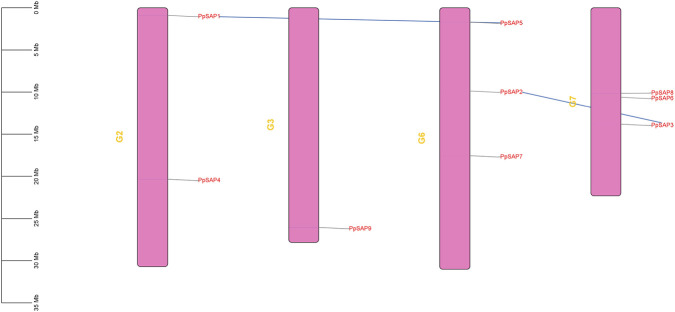
Chromosomal distribution of *PpSAPs* and intragenomic collinearity. Physical positions of the nine *PpSAPs* on peach chromosomes are shown. Intragenomic collinear links connect duplicated *PpSAP* gene pairs.

**FIGURE 5:**
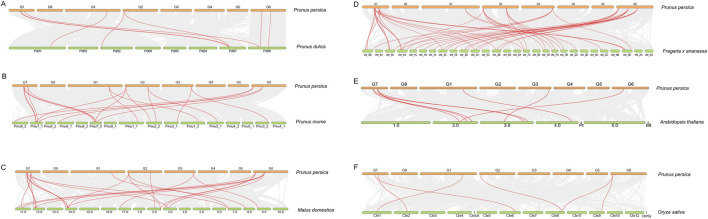
Interspecies synteny of *PpSAP1-PpSAP9* loci between *Prunus persica* and six representative plant genomes. **(A)** *Prunus persica* vs. *Prunus dulcis*; **(B)** *Prunus persica* vs. *Prunus mume*; **(C)** *Prunus persica* vs. *Malus domestica*; **(D)** *Prunus persica* vs. *Fragaria* × *ananassa*; **(E)** *Prunus persica* vs. *Arabidopsis thaliana*; and **(F)** *Prunus persica* vs. *Oryza sativa*. Colored bars represent chromosomes (or pseudochromosomes) of each species, with different colors denoting different chromosomes. Red links indicate syntenic orthologous gene pairs or collinear blocks involving *PpSAP1-PpSAP9* identified between *P. persica* and the corresponding species. Syntenic blocks were detected using MCScanX implemented in TBtools (v2.376) with an E-value cutoff of 1e^−10^, and dual-synteny visualization was generated using the TBtools Dual Synteny Plot module. The plots show the whole-chromosome frameworks for both species, whereas the links are restricted to *SAP*-associated syntenic relationships anchored by *PpSAP1-PpSAP9*.

To investigate the genomic organization and evolutionary expansion of the peach SAP family, the nine *PpSAPs* (*PpSAP1-PpSAP9*) were mapped onto the *Prunus persica* genome and analyzed using intragenomic collinearity together with interspecies synteny comparisons (Figures 4, 5). The nine loci were unevenly distributed across four chromosomes (G2, G3, G6, and G7; Figure 4). Chromosomes G6 and G7 each contained three *PpSAPs*, whereas two genes were located on G2 and one gene on G3, indicating a non-random genomic distribution that may reflect differential retention of SAP-associated genomic segments.

Intragenomic collinearity analysis identified two WGD/segmental duplication-derived gene pairs within the *PpSAP* family: *PpSAP1–PpSAP5* and *PpSAP2–PpSAP3* (Figure 4). Consistent with MCScanX duplication-type annotation, these genes were classified as “WGD/segmental,” supporting their origin from large-scale duplication events. Ka/Ks ratios were 0.134 for *PpSAP1-PpSAP5* and 0.228 for *PpSAP2-PpSAP3* (Supplementary Table S1), both well below 1, indicating predominant purifying selection during post-duplication evolution. By contrast, many other pairwise comparisons showed substantial synonymous-site saturation (pS > 0.75) or undefined Ks values, resulting in NA Ka/Ks estimates; these comparisons were therefore retained for describing divergence but excluded from selective-pressure inference (Supplementary Table S1).

To assess the evolutionary conservation of *PpSAP* loci, we performed interspecies synteny analyses between *Prunus persica* and six representative species, including *Prunus dulcis* (Figure 5A), *Prunus mume* (Figure 5B), *Malus domestica* (Figure 5C), *Fragaria × ananassa* (Figure 5D), *Arabidopsis thaliana* (Figure 5E), and *Oryza sativa* (Figure 5F). Focusing on *PpSAP1-PpSAP9*, a total of 100 non-redundant syntenic orthologous pairs were identified across the six comparisons (Supplementary Table S2). The number of syntenic pairs showed a clear phylogenetic gradient, with markedly higher counts within Rosaceae (42 pairs with *F.* × *ananassa*, 22 with *M. domestica*, 17 with *P. mume*, and 7 with *P. dulcis*) than in more distant taxa (7 with *A. thaliana* and 5 with *O. sativa*), indicating stronger conservation of *PpSAP*-associated genomic contexts within Rosaceae and only residual retention across deep angiosperm divergence.

Within Rosaceae, *F.* × *ananassa* exhibited the most extensive one-to-many correspondences (Figure 5D), consistent with multi-subgenome homeolog retention in this polyploid genome. Several peach genes displayed relatively high connectivity, notably *PpSAP3*, followed by *PpSAP1*, *PpSAP2*, and *PpSAP6*. In *M. domestica*, multi-copy correspondences were also observed, whereas *P. mume* and *P. dulcis* showed fewer but still clear orthologous relationships with peach *PpSAP* loci. In contrast, syntenic links with *A. thaliana* and *O. sativa* were sparse, although several conserved orthologous connections remained detectable, supporting an ancient origin of *SAP*-associated genomic regions followed by lineage-specific retention and divergence.

### 3.5 Cis-acting regulatory elements in *PpSAP* promoters

**FIGURE 6:**
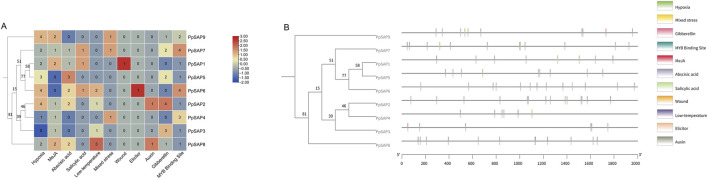
Cis-acting regulatory elements in the 2-kb promoter regions of *PpSAPs*. **(A)** Heatmap summarizing the counts of major stress- and hormone-responsive cis-elements within each 2-kb promoter region upstream of the start codon (ATG). **(B)** Positional distribution of these cis-elements across the 0–2 kb promoter intervals, highlighting representative categories including ABA, GA, low-temperature, wound, and SA-responsive elements, MYB-binding sites, and anaerobic/anoxic response elements.

Scanning of the 2-kb upstream promoter regions of the nine *PpSAPs* revealed a diverse cis-regulatory landscape enriched in both hormone- and stress-responsive elements (Figure 6). In the element-count heatmap, motifs associated with hormone responses, including ABA, MeJA, and SA-related elements, and abiotic stress responses, including low-temperature, wound, and anaerobic/anoxic-responsive elements, were widely detected across the family. However, the abundance and combinations of motif categories varied markedly among individual promoters, indicating substantial promoter-level diversification within the *PpSAP* family (Figure 6A).

Hierarchical clustering based on motif counts separated *PpSAP* promoters into distinct groups with contrasting regulatory signatures (Figure 6A). One group exhibited a relatively higher representation of anaerobic/anoxic response elements together with multiple hormone-responsive motifs, whereas another group contained fewer anaerobic elements but retained a broader mixture of hormone- and stress-associated motifs. MYB-binding sites were detected in multiple promoters and contributed prominently to between-gene differences in the heatmap, suggesting that MYB-associated regulation may represent an important component of *PpSAP* transcriptional control.

Consistent with the count-based summary, positional mapping showed that cis-elements were distributed throughout the 0–2000 bp promoter intervals, with several motif categories occurring in both proximal and distal regions (Figure 6B). Stress- and hormone-responsive elements frequently co-occurred within the same promoter, indicating that *PpSAP* transcription may integrate inputs from multiple signaling pathways. The marked variation in the number and composition of these cis-elements further suggests that different *PpSAPs* may be regulated by distinct combinations of stress and hormone signaling pathways. Together, these quantitative and positional analyses indicate that *PpSAP* promoters exhibit heterogeneous yet multi-input regulatory architectures, providing a cis-regulatory basis for the divergent transcriptional responses observed under abiotic stresses and hormone-related treatments.

### 3.6 Expression profiles of *PpSAPs* under different abiotic stress treatments

**FIGURE 7:**
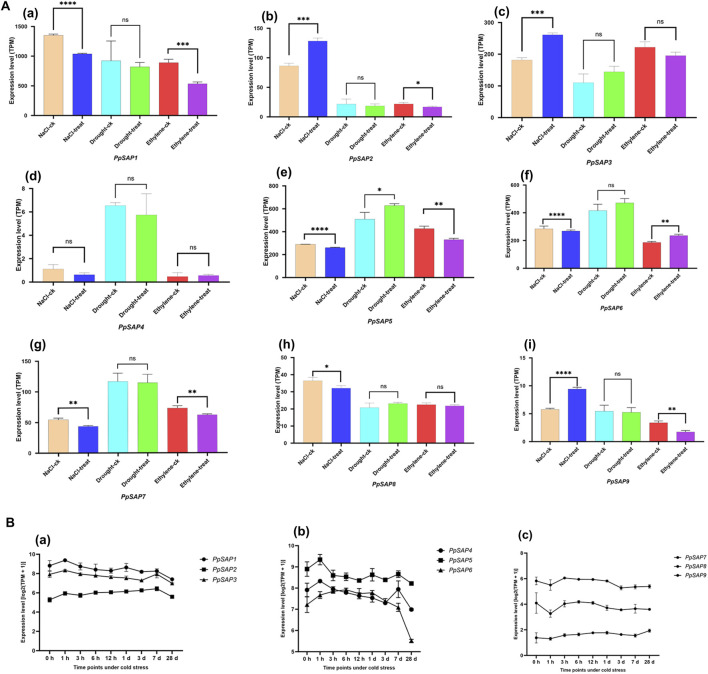
Expression profiles of *PpSAPs* under different abiotic stress treatments based on RNA-seq data. **(A)** Expression levels of *PpSAP1-PpSAP9* under NaCl, drought, and ethylene treatments. The y-axis indicates expression level (TPM). Bars represent the mean ± SD of three biological replicates. Significant differences between each treatment and its corresponding control are indicated by asterisks (*, p < 0.05; **, p < 0.01; ***, p < 0.001; ****, p < 0.0001; ns, not significant). **(B)** Time-course expression patterns of *PpSAP1-PpSAP9* under cold stress. The y-axis indicates expression level [log2 (TPM + 1)]. Data are presented as mean ± SD of three biological replicates. The x-axis represents time points under cold stress (0 h, 1 h, 3 h, 6 h, 12 h, 1 d, 3 days, 7 days, and 28 days).

To investigate the potential roles of *PpSAPs* in abiotic stress responses, RNA-seq data were used to analyze their expression patterns under NaCl, drought, ethylene, and cold stress conditions (Figure 7). Under NaCl, drought, and ethylene treatments, the nine *PpSAPs* exhibited distinct transcriptional responses relative to their corresponding controls, and several members showed significant expression changes (Figure 7A), indicating differential responsiveness of the *PpSAP* family to different stress-related signals.

Under cold stress, the expression patterns of *PpSAPs* showed obvious temporal variation across different treatment durations (Figure 7B). *PpSAP1*, *PpSAP2*, and *PpSAP9* displayed relatively rapid responses at the early stage of treatment, with transcript levels reaching higher levels at 1 h and then generally declining during prolonged cold exposure. *PpSAP8* also showed an early-to-middle stage increase, reaching a relatively high level between 3–6 h, followed by a marked decrease at later time points, especially at 28 days. In contrast, *PpSAP3* showed a gradual increase during cold treatment and reached its highest level at 7 days, whereas *PpSAP6* and *PpSAP7* exhibited relatively low transcript abundance but showed a progressive increase during prolonged cold exposure, with the highest levels observed at 28 d. *PpSAP4* remained at a relatively low expression level throughout the treatment period with only minor fluctuations, while *PpSAP5* peaked at the early stage and then decreased gradually after extended cold treatment.

Taken together, these results indicate that *PpSAPs* exhibit diverse and stress-dependent expression profiles. The distinct temporal responses observed under cold stress further suggest potential functional divergence among *PpSAP* family members in peach abiotic stress adaptation.

## 4 Discussion

### 4.1 Evolutionary retention and structural diversification of *PpSAPs*

The identification of nine *PpSAPs* in peach, together with their classification into four phylogenetic clades, supports the view that the SAP family is evolutionarily retained across angiosperms while also showing lineage-dependent diversification. In peach, the SAP family exhibited stronger conservation with SAPs from other Rosaceae species, such as *Malus domestica* and *Prunus mume*, whereas conservation was weaker in more distantly related species such as *Arabidopsis thaliana* and *Oryza sativa*. This pattern is consistent with the reduced number of syntenic gene pairs detected in distant taxa and suggests that SAP-associated genomic regions have been preferentially retained within Rosaceae, while deeper evolutionary divergence has been accompanied by more extensive remodeling.

The structural diversity observed within the PpSAP family, including A20-AN1, AN1-only, and AN1 × 2 types, further highlights the evolutionary complexity of this gene family. In particular, PpSAP8 and PpSAP9 occupy relatively divergent positions in the phylogenetic and structural analyses, and their distinct motif organization suggests greater subgroup-specific specialization than that observed for more conserved family members. Moreover, promoter and expression analyses indicate that these genes may have developed lineage-specific regulatory features, potentially linked to distinct stress signaling pathways. Similar promoter-level divergence has also been reported in other Rosaceae-related systems, further supporting the possibility of functional differentiation among SAP family members (Lloret et al., 2017).

### 4.2 SAPs as regulators in abiotic stress responses: links to the ubiquitin-proteasome pathway

Many A20/AN1-type SAPs have been reported to display E3 ligase activity and to participate in stress-responsive pathways through the ubiquitin–proteasome system (Kang et al., 2011; Zhang et al., 2017). For example, in wheat, TaSAP5 promotes the degradation of DRIP proteins and enhances drought tolerance by stabilizing DREB2A (Zhang et al., 2017). Similarly, in orchids, Pha13, a homolog of AtSAP5, functions in salicylic acid-mediated immunity via E3 ligase activity (Chang et al., 2018). These findings suggest that SAP proteins can act as important modulators of stress-related signaling networks rather than simply as passive stress markers.

However, not all SAPs have been experimentally confirmed to exhibit E3 ligase activity. In peach, structurally divergent members, especially AN1-only and AN1 × 2 proteins such as PpSAP8 and PpSAP9, may participate in stress responses through protein–protein interactions or as components of regulatory complexes rather than through direct ubiquitination. Although these possibilities remain to be experimentally verified, similar interpretations have been proposed for SAP proteins in other systems (Dixit et al., 2018). The structural divergence of these peach SAPs, together with their distinct expression patterns under stress, supports the idea that different PpSAP members may contribute unequally to abiotic stress-related pathways.

### 4.3 Cis-regulatory diversification as a basis for differential transcriptional responsiveness

Promoter analysis revealed that *PpSAP* promoters contain a rich repertoire of cis-elements associated with stress and hormone signaling, including ABA-responsive motifs, MYB-binding sites, and elements related to cold, salt, and anaerobic/anoxic responses. This promoter heterogeneity suggests that different *PpSAPs* may be controlled by distinct combinations of environmental and hormonal signals rather than functioning within a single uniformly regulated transcriptional module. The clustering of *PpSAP* promoters based on motif composition further supports the idea that these genes participate in different regulatory subnetworks.

The presence of MYB-binding sites in several promoters is particularly noteworthy, as MYB transcription factors are widely implicated in abiotic stress and hormone signaling. This suggests that MYB-mediated regulation may represent one important route through which *PpSAPs* are transcriptionally modulated. Similar promoter diversity has also been reported in other species, such as Medicago truncatula and Prunus dulcis, where SAP family members exhibit diverse cis-regulatory architectures (Fatima et al., 2022; Zhou et al., 2018). Taken together, these findings provide a plausible cis-regulatory basis for the divergent transcriptional responses observed among *PpSAPs* under abiotic stress and hormone-related treatments.

### 4.4 Divergent stress-responsive expression patterns of *PpSAPs* and comparison with other crops

Re-analysis of RNA-seq data showed that the *PpSAPs* exhibit diverse expression patterns in response to abiotic stresses and hormone treatment. Several genes showed significant expression changes under NaCl, drought, and ethylene treatments, whereas others showed weaker or more limited responses, indicating that the *PpSAP* family is not uniformly co-regulated but instead displays clear transcriptional diversification (Dixit et al., 2018). This overall pattern suggests that different *PpSAPs* may participate in distinct stress- and hormone-related regulatory pathways.

The cold-stress time-course analysis further revealed clear temporal divergence among family members. *PpSAP1*, *PpSAP2*, and *PpSAP9* responded relatively rapidly at early stages of treatment, whereas *PpSAP8* showed an early-to-middle stage increase followed by a decline at later time points. In contrast, *PpSAP3* exhibited a more gradual increase and reached its highest expression at a later stage, while *PpSAP6* and *PpSAP7* showed relatively low transcript abundance but displayed delayed accumulation during prolonged cold exposure. These contrasting temporal patterns suggest that different *PpSAPs* may function at different phases of stress response, including early signaling, intermediate adjustment, and longer-term acclimation.

These findings are broadly consistent with results reported in other species. Within the Rosaceae family, stress-responsive expression of *SAP* genes has also been observed in Malus domestica and Prunus dulcis, supporting the view that SAP family members may play conserved but non-identical roles in stress adaptation across related fruit crops (Dong et al., 2018; Fatima et al., 2022). In other crops, including wheat, apple, soybean, and maize, SAP genes have likewise been implicated in responses to drought, salinity, and other abiotic stresses, although their expression patterns remain gene- and species-dependent (Fu et al., 2022; Li et al., 2019; Vij and Tyagi, 2006; Zhang et al., 2019; Zhao et al., 2021; Zhou et al., 2018). Thus, although SAP genes retain conserved core features across plants, their expression outputs and regulatory roles appear to have diversified according to species background and physiological requirements.

### 4.5 Implications for peach stress resilience and breeding-oriented applications

Although CRISPR-based functional validation of these genes remains a promising long-term strategy, the relatively long functional timeline in peach makes short-cycle approaches especially valuable. Techniques such as transient expression in Arabidopsis or tobacco, or virus-induced gene silencing (VIGS) in peach, may provide practical tools for early functional studies (Burch-Smith et al., 2004). These approaches could facilitate rapid assessment of candidate gene functions in stress-response pathways without requiring long-term genomic modification.

From an applied perspective, the present study does not by itself establish direct breeding utility, but it does provide a useful framework for prioritizing *PpSAP* candidates for future functional analysis. Genes with clear stress-responsive expression patterns and divergent structural or regulatory features may be especially informative for further validation. Such work will help clarify how different *PpSAPs* contribute to peach adaptation to abiotic stress and may ultimately support breeding-related research aimed at improving stress resilience in peach cultivation.

## 5 Conclusion

In conclusion, this study provides the first comprehensive characterization of the SAP gene family in peach (*Prunus persica*). A total of nine *PpSAPs* were identified and classified into four phylogenetic clades, revealing both evolutionary conservation and lineage-specific diversification of *PpSAPs* in peach. Integrated analyses of phylogeny, domain and motif organization, gene structure, chromosomal distribution, duplication patterns, and interspecies synteny indicated that the PpSAP family has retained conserved core features while also developing distinct structural characteristics in specific members.

Promoter analysis showed that *PpSAPs* contain abundant cis-regulatory elements related to stress and hormone signaling, providing a regulatory basis for their differential expression. RNA-seq profiling further demonstrated that *PpSAPs* exhibit diverse transcriptional responses under NaCl, drought, ethylene, and cold stress conditions, with clear temporal divergence observed during cold treatment. These results support the view that different PpSAP members may contribute unequally to peach abiotic stress responses.

Overall, this study establishes a valuable genomic and evolutionary framework for the *PpSAP* family and identifies several stress-responsive candidate genes for future functional validation. These findings provide a useful foundation for further research on peach stress adaptation and may support breeding-related efforts aimed at improving stress resilience in peaches.

## Data Availability

The raw contributions presented in the study are publicly available. These data can be found here: Zenodo, https://doi.org/10.5281/zenodo.18766288.

## Supplementary material

SUPPLEMENTARY TABLE S1 Selection pressure analysis of PpSAP gene pairs.

SUPPLEMENTARY TABLE S2 Syntenic orthologous gene pairs of PpSAP1-PpSAP9 between Prunus persica and six representative plant genomes.

SUPPLEMENTARY TABLE S3 Stress-related TPM values of peach genes under abiotic stress treatments.

SUPPLEMENTARY TABLE S4 Public RNA-seq libraries used for expression profiling of PpSAPs in Prunus persica.

SUPPLEMENTARY FIGURE S1 Heatmap of PpSAPs gene expression across abiotic stress conditions. Values are shown as TPM means from three biological replicates. Genes are clustered based on hierarchical clustering of expression patterns, and treatments include NaCl, drought, ethylene, and cold stress.

## References

[B1] Bailey T. L. Johnson J. Grant C. E. Noble W. S. (2015). The MEME suite. Nucleic Acids Res. 43 (W1), W39–W49. 10.1093/nar/gkv416 25953851 PMC4489269

[B2] Ben Saad R. Farhat-Khemekhem A. Ben Halima N. Ben Hamed K. Brini F. Saibi W. (2018). The LmSAP gene isolated from the halotolerant Lobularia maritima improves salt and ionic tolerance in transgenic tobacco lines. Funct. Plant Biol. 45 (3), 378–391. 10.1071/FP17202 32290960

[B3] Ben Saad R. Safi H. Ben Hsouna A. Brini F. Ben Romdhane W. (2019). Functional domain analysis of LmSAP protein reveals the crucial role of the zinc-finger A20 domain in abiotic stress tolerance. Protoplasma 256 (5), 1333–1344. 10.1007/s00709-019-01390-2 31062172

[B4] Berardini T. Z. Reiser L. Li D. Mezheritsky Y. Muller R. Strait E. (2015). The arabidopsis information resource: making and mining the “gold standard” annotated reference plant genome. Genesis 53 (8), 474–485. 10.1002/dvg.22877 26201819 PMC4545719

[B5] Burch-Smith T. M. Anderson J. C. Martin G. B. Dinesh-Kumar S. P. (2004). Applications and advantages of virus-induced gene silencing for gene function studies in plants. Plant J. 39 (Number 5), 734–746. 10.1111/j.1365-313X.2004.02158.x 15315635

[B33] Camacho C. Coulouris G. Avagyan V. Ma N. Papadopoulos J. Bealer K. (2009). BLAST+: architecture and applications. BMC Bioinform. 10, 421. 10.1186/1471-2105-10-421 20003500 PMC2803857

[B6] Chang L. Chang H. H. Chang J. C. Lu H. C. Wang T. T. Hsu D. W. (2018). Plant A20/AN1 protein serves as the important hub to mediate antiviral immunity. PLoS Pathog. 14 (9), e1007288. 10.1371/journal.ppat.1007288 30212572 PMC6155556

[B7] Chen C. Wu Y. Li J. Wang X. Zeng Z. Xu J. (2023). TBtools-II: a “one for all, all for one” bioinformatics platform for biological big-data mining. Mol. Plant 16 (11), 1733–1742. 10.1016/j.molp.2023.09.010 37740491

[B8] Dixit A. Tomar P. Vaine E. Abdullah H. Hazen S. Dhankher O. P. (2018). A stress-associated protein, AtSAP13, from *Arabidopsis thaliana* provides tolerance to multiple abiotic stresses. Plant Cell Environ. 41 (5), 1171–1185. 10.1111/pce.13103 29194659

[B9] Dong Q. Duan D. Zhao S. Xu B. Luo J. Wang Q. (2018). Genome-wide analysis and cloning of the apple stress-associated protein gene family reveals *MdSAP15*, which confers tolerance to drought and osmotic stresses in transgenic Arabidopsis. Int. J. Mol. Sci. 19 (9), 2478. 10.3390/ijms19092478 30134640 PMC6164895

[B10] Fatima S. Zafar Z. Gul A. Bhatti M. F. (2022). Genome-wide identification of stress-associated proteins (Saps) encoding a20/an1 zinc finger in almond (prunus dulcis) and their differential expression during fruit development. Plants 11 (1), 117. 10.3390/plants11010117 35009120 PMC8747467

[B11] Fu Q. Duan H. Cao Y. Li Y. Lin X. L. Pang H. (2022). Comprehensive identification and functional analysis of stress-associated protein (SAP) genes in osmotic stress in maize. Int. J. Mol. Sci. 23 (22), 14010. 10.3390/ijms232214010 36430489 PMC9692755

[B12] Kang M. Fokar M. Abdelmageed H. Allen R. D. (2011). Arabidopsis SAP5 functions as a positive regulator of stress responses and exhibits E3 ubiquitin ligase activity. Plant Mol. Biol. 75 (4–5), 451–466. 10.1007/s11103-011-9748-2 21293909

[B34] Lescot M. Déhais P. Thijs G. Marchal K. Moreau Y. Van de Peer Y. (2002). PlantCARE, a database of plant cis-acting regulatory elements and a portal to tools for in silico analysis of promoter sequences. Nucleic Acids Res. 30 (1), 325–327. 10.1093/nar/30.1.325 11752327 PMC99092

[B13] Letunic I. Bork P. (2021). Interactive tree of life (iTOL) v5: an online tool for phylogenetic tree display and annotation. Nucleic Acids Res. 49 (W1), W293–W296. 10.1093/nar/gkab301 33885785 PMC8265157

[B14] Li J. Sun P. Xia Y. Zheng G. Sun J. Jia H. (2019). A stress-associated protein, *PtSAP13*, from *Populus trichocarpa* provides tolerance to salt stress. Int. J. Mol. Sci. 20 (22), 5782. 10.3390/ijms20225782 31744233 PMC6888306

[B15] Lian X. Zhang H. Jiang C. Gao F. Yan L. Zheng X. (2022). *De novo* chromosome-level genome of a semi-dwarf cultivar of Prunus persica identifies the aquaporin PpTIP2 as responsible for temperature-sensitive semi-dwarf trait and PpB3-1 for flower type and size. Plant Biotechnol. J. 20 (5), 886–902. 10.1111/pbi.13767 34919780 PMC9055816

[B16] Liu J. Yang X. Yang X. Xu M. Liu J. Xue M. (2017). Isolation and characterization of LcSAP, a Leymus chinensis gene which enhances the salinity tolerance of *Saccharomyces cerevisiae* . Mol. Biol. Rep. 44 (1), 5–9. 10.1007/s11033-016-4091-y 27853974

[B17] Lloret A. Conejero A. Leida C. Petri C. Gil-Muñoz F. Burgos L. (2017). Dual regulation of water retention and cell growth by a stress-associated protein (SAP) gene in prunus. Sci. Rep. 7 (1), 332. 10.1038/s41598-017-00471-7 28336950 PMC5428470

[B18] Lu S. Wang J. Chitsaz F. Derbyshire M. K. Geer R. C. Gonzales N. R. (2020). CDD/SPARCLE: the conserved domain database in 2020. Nucleic Acids Res. 48 (D1), D265-D268. 10.1093/nar/gkz991 31777944 PMC6943070

[B19] Minh B. Q. Nguyen M. A. T. Von Haeseler A. (2013). Ultrafast approximation for phylogenetic bootstrap. Mol. Biol. Evol. 30 (5), 1188–1195. 10.1093/molbev/mst024 23418397 PMC3670741

[B20] Mistry J. Chuguransky S. Williams L. Qureshi M. Salazar G. A. Sonnhammer E. L. L. (2021). Pfam: the protein families database in 2021. Nucleic Acids Res. 49 (D1), D412–D419. 10.1093/nar/gkaa913 33125078 PMC7779014

[B21] Ødum M. T. Teufel F. Thumuluri V. Armenteros J. J. A. Johansen A. R. Winther O. (2024). DeepLoc 2.1: multi-label membrane protein type prediction using protein language models. Nucleic Acids Res. 52 (W1), W215–W220. 10.1093/nar/gkae237 38587188 PMC11223819

[B22] Parra S. Núñez-Lillo G. Tapia-Reyes P. Carrasco-Lozano E. C. Porcile V. Gonzalez-Calquin C. (2025). Decoding the Hayward kiwi (Actinidia deliciosa var Hayward) genome: transcriptomic responses to drought and salinity and AdhSAP4’s role in salinity stress responses. Front. Plant Sci. 16, 1637092. 10.3389/fpls.2025.1637092 41064751 PMC12500665

[B23] Robert X. Gouet P. (2014). Deciphering key features in protein structures with the new ENDscript server. Nucleic Acids Res. 42 (W1), W320–W324. 10.1093/nar/gku316 24753421 PMC4086106

[B24] Thumuluri V. Almagro Armenteros J. J. Johansen A. R. Nielsen H. Winther O. (2022). DeepLoc 2.0: multi-label subcellular localization prediction using protein language models. Nucleic Acids Res. 50 (W1), W228–W234. 10.1093/nar/gkac278 35489069 PMC9252801

[B25] Vashisth V. Sharma G. Giri J. Sharma A. K. Tyagi A. K. (2024). Rice A20/AN1 protein, OsSAP10, confers water-deficit stress tolerance *via* proteasome pathway and positive regulation of ABA signaling in arabidopsis. Plant Cell Rep. 43 (9), 215. 10.1007/s00299-024-03304-w 39138747

[B26] Vij S. Tyagi A. K. (2006). Genome-wide analysis of the stress associated protein (SAP) gene family containing A20/AN1 zinc-finger(s) in rice and their phylogenetic relationship with arabidopsis. Mol. Genet. Genomics 276 (6), 565–575. 10.1007/s00438-006-0165-1 17033811

[B27] Wang Y. Tang H. Debarry J. D. Tan X. Li J. Wang X. (2012). MCScanX: a toolkit for detection and evolutionary analysis of gene synteny and collinearity. Nucleic Acids Res. 40 (7), e49. 10.1093/nar/gkr1293 22217600 PMC3326336

[B28] Yoon S. K. Bae E. K. Lee H. Choi Y. I. Han M. Choi H. (2018). Downregulation of stress-associated protein 1 (PagSAP1) increases salt stress tolerance in poplar (Populus alba × P. glandulosa). Trees - Struct. Funct. 32 (3), 823–833. 10.1007/s00468-018-1675-2

[B29] Zhang N. Yin Y. Liu X. Tong S. Xing J. Zhang Y. (2017). The E3 ligase TaSAP5 alters drought stress responses by promoting the degradation of DRIP proteins. Plant Physiol. 175 (4), 1878–1892. 10.1104/pp.17.01319 29089392 PMC5717742

[B30] Zhang X. Z. Zheng W. J. Cao X. Y. Cui X. Y. Zhao S. P. Yu T. F. (2019). Genomic analysis of stress associated proteins in soybean and the role of GmSAP16 in abiotic stress responses in arabidopsis and soybean. Front. Plant Sci. 10, 1453. 10.3389/fpls.2019.01453 31803204 PMC6876671

[B31] Zhao X. Wang R. Zhang Y. Li Y. Yue Y. Zhou T. (2021). Comprehensive analysis of the stress associated protein (SAP) gene family in Tamarix hispida and the function of ThSAP6 in salt tolerance. Plant Physiology Biochem. 165, 1–9. 10.1016/j.plaphy.2021.05.016 34029940

[B32] Zhou Y. Zeng L. Chen R. Wang Y. Song J. (2018). Genome-wide identification and characterization of stress-associated protein (SAP) gene family encoding A20/AN1 zinc-finger proteins in Medicago truncatula. Archives Biol. Sci. 70 (1), 87–98. 10.2298/ABS170529028Z

