# Supplementary figures and images for: Comparative genomic and evolutionary analysis of stress-associated proteins in Prunus persica and distinct plant species under abiotic stress
Source: Front Genet. 2026 May 12;17:1821359. doi: 10.3389/fgene.2026.1821359 (PMC13200825; doi:10.3389/fgene.2026.1821359)

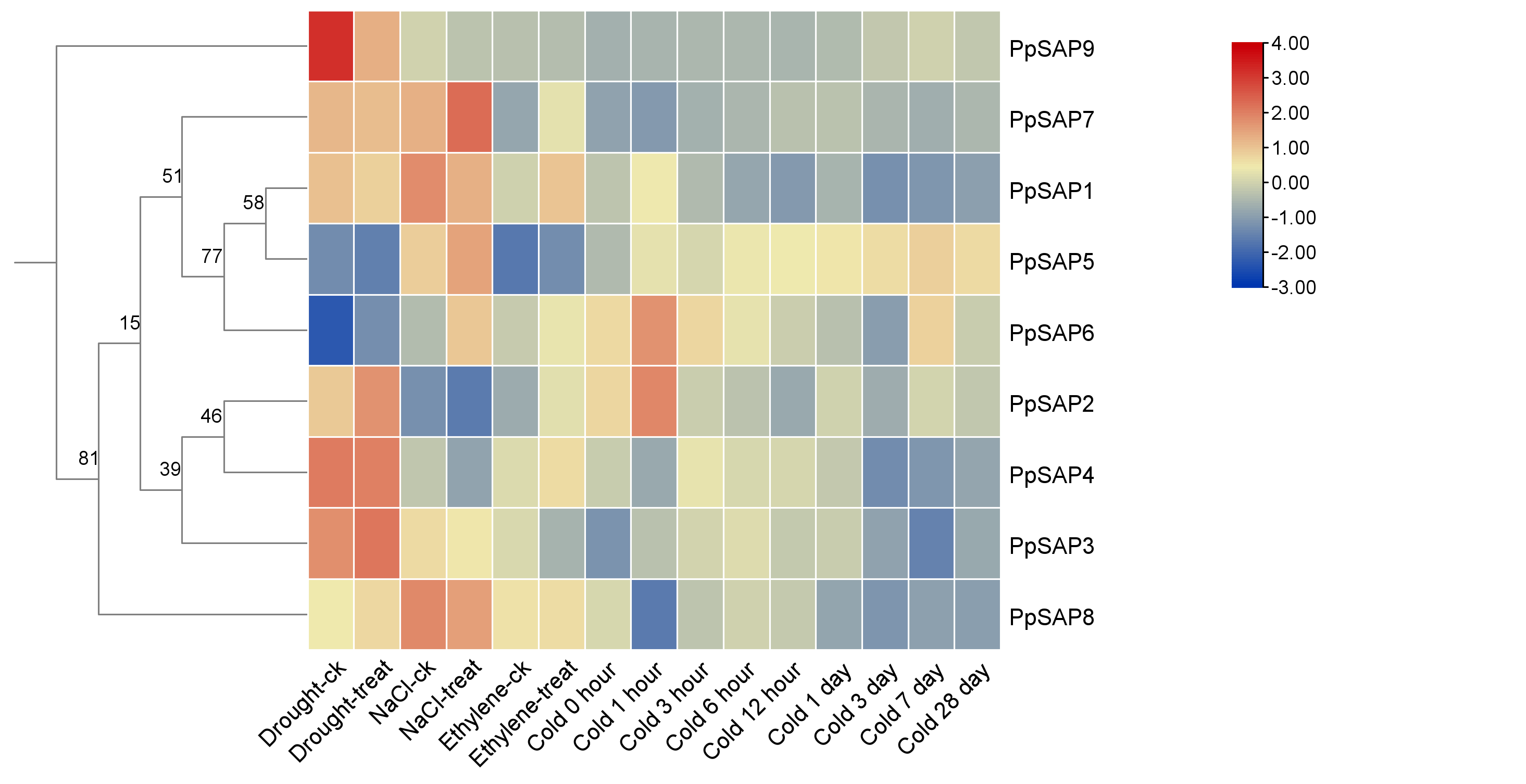

Supplement: Supplementary file 2 [file Image1.tiff]
